# Occupational Exposure to Incidental Nanomaterials in Metal Additive Manufacturing: An Innovative Approach for Risk Management

**DOI:** 10.3390/ijerph20032519

**Published:** 2023-01-31

**Authors:** Marta Sousa, Pedro Arezes, Francisco Silva

**Affiliations:** 1 ALGORITMI Research Center/LASI, University of Minho, 4800-058 Guimarães, Portugal; 2 CATIM—Technological Center for the Metal Working Industry, 4100-414 Porto, Portugal; 3 CTCV—Technological Center for Ceramic and Glass, 3040-540 Coimbra, Portugal

**Keywords:** incidental nanoparticles, control banding, risk management, occupational exposure, metal additive manufacturing

## Abstract

The benefits of metal 3D printing seem unquestionable. However, this additive manufacturing technology brings concerns to occupational safety and health professionals, since recent studies show the existence of airborne nanomaterials in these workplaces. This article explores different approaches to manage the risk of exposure to these incidental nanomaterials, on a case study conducted in a Portuguese organization using Selective Laser Melting (SLM) technology. A monitoring campaign was performed using a condensation particle counter, a canning mobility particle sizer and air sampling for later scanning electron microscopy and energy dispersive X-ray analysis, proving the emission of nano-scale particles and providing insights on number particle concentration, size, shape and chemical composition of airborne matter. Additionally, Control Banding Nanotool v2.0 and Stoffenmanager Nano v1.0 were applied in this case study as qualitative tools, although designed for engineered nanomaterials. This article highlights the limitations of using these quantitative and qualitative approaches when studying metal 3D Printing workstations. As a result, this article proposes the IN Nanotool, a risk management method for incidental nanomaterials designed to overcome the limitations of other existing approaches and to allow non-experts to manage this risk and act preventively to guarantee the safety and health conditions of exposed workers.

## 1. Introduction

Freedom of design, time efficiency, reduction of labor and machine costs are a few examples of the several advantages mentioned when the subject is metal 3D Printing, also known as metal Additive Manufacturing (AM) [1]. Regardless of its considerable potential, metal AM has been raising some concerns regarding occupational health and safety [2]. Among other occupational risks, it is known that during these processes incidental metal nano-objects are emitted and it is essential to manage the risk of exposure to this airborne matter to reduce possible negative ill-health effects on workers [3].

Different approaches have been used to assess and/or manage the occupational risk of exposure to incidental nanomaterials in AM processes, but the definition of standardized methods still remains an urgent need [4]. Looking at this occupational risk from the point of view of the common industrial hygiene approach, it is possible to monitor and to quantify the airborne matter released during metal 3D printing. Recent publications in this field endorse the use of direct-reading instruments (for example condensation particle counter—CPC, optical particle counter—OPC and scanning mobility particle sizer—SMPS) and/or the collection of samples and subsequent structural and chemical analysis, by using scanning electron microscopy (SEM), transmission electron microscopy (TEM) and/or energy dispersive X-ray analyzers (EDS) [2,3,5,6,7,8]. However, this attempt at a more industrial hygiene conservative approach has limitations that cross all these studies: The lack of clearly defined and standardized occupational exposure limits for metal incidental nanomaterials and the lack of standardized sampling strategies. Some of these studies use as comparison reference values for nanomaterials proposed by different competent local entities and institutes, but so far, no specific limits have been proposed for metal incidental nanomaterials. The most common approach is to compare the results to the recommended benchmarks defined by the Nanosafety Research Centre of the Finnish Institute of Occupational Health (FIOH), i.e., 20,000 nanoparticles/cm^3^ (with a density higher than 6000 kg/m^3^) for an 8-h exposure time. This limit was later adopted by the Institute for Occupational Safety and Health of the German Social Accident Insurance (IFA DGUV) and the IVAM Environmental Research UVA BV in the Netherlands [5,9]. Even if this value is assumed to be an appropriate reference for metal AM case studies, the quantitative risk assessment still has limitations, namely the possible lack of access to equipment and laboratory analysis for these monitoring campaigns and also the lack of experts to perform them and interpret the results.

Another possibility to assess this risk during metal AM has been to apply qualitative methods originally designed for engineered nanomaterials (ENM), namely control banding based ones. Sousa et al. [8] and Dugheri et al. [5] applied Control Banding Nanotool v2.0 to assess the risk of exposure to ultrafine particles during metal 3D printing operations. Sousa et al. [8] highlight some difficulties on using this approach for incidental nanoparticles, especially the lack of background information on the particles (such as size, shape, and solubility, among others). These authors suggest the design of new methods for incidental nanomaterials, with different inputs than the ones for ENM, to reduce the uncertainty associated with the assessment. Dugheri et al. [5] also emphasize the importance of searching different strategies to assess this occupational risk.

This article aims to explore different approaches to study the potential exposure to incidental nanomaterials during metal AM, through a case study conducted in an organization using Selective Laser Melting (SLM) technology. The main purpose of this article is to propose a risk management tool, entitled IN Nanotool, designed for incidental metal nanomaterials originated from metal AM processes to overcome the limitations of other existing approaches.

## 2. Materials and Methods

### 2.1. Facility, Operation Conditions and Materials

This study was conducted in an organization that uses Selective Laser Melting (SLM) technology for metal additive manufacturing. The SLM printer is located in a room dedicated to prototyping, with approximately 85 m^2^ and 3 m in height. On the data collection day, no other equipment besides the printer was operating.

The printing process consists in the deposition of layers of a metal powder, usually 20 to 70 microns depending on materials, followed by the application of an infrared laser light scan (1064 nm) of 250 W that melts the powder to reproduce a three-dimensional part, previously defined in a CAD program. The material used was a nitrogen gas atomized spherical powder for additive manufacturing: Stainless steel 316L, with particle size between 20 and 53 μm. Stainless steel 316L is an alloy of iron (>75%) and chromium (≈17%) which also contains nickel (≈12.5%), molybdenum (≈2.5%) and other elements in less significant amount. In this case study, 59.15 cm^3^ of this powder were used during the printing process but the final part only had 0.35 cm^3^ (approximately 0.59%).

In addition to the initial preparation for printing (which includes CAD design and filling the powder in the printer), the worker’s tasks can be divided into 3 distinct phases, as described in Table 1.

Data gathering included a sample of powder before and after the printing process, technical and material safety data sheets of the powder, information on operation conditions and on-site measurements.

### 2.2. Quantitative Approach

In the attempt to study the risk of exposure to airborne nanomaterials using a quantitative assessment, the following equipment was used:

- A thermo-hygrometer, TSI^®^ Model 9545 (TSI Incorporated, MN, USA), to measure air velocity, room temperature and relative humidity.
- Portable condensation particle counter (CPC), TSI^®^ model 3007, to measure the total particle number concentration from 10 nm to >1000 nm in 1-s time resolution.
- A scanning mobility particle sizer (SMPS), TSI^®^ Model 3910, to measure nanoparticle size distributions and concentrations, with a size distribution from 10 to 420 nm. The number of particles per size was measured by an internal CPC which counts single particles to provide accurate counts, even at low concentrations.
- A personal air sampling pump, SKC AirChek^®^ TOUCH (SKC, PA, USA), to collect samples for subsequent Scanning Electron Microscopy (SEM) and Energy-dispersive X-ray spectroscopy (EDS) analysis. The samples were collect using a polycarbonate membrane filter (with 25 mm diameter and 0.4 μm porosity) and a heat-treated quartz filter (DPM Cassette with 0.8 µm Impactor), since these types of filters were used in previous studies and proved to be effective for nanomaterials [10,11,12].

The monitoring campaign started with background measurements before any printing activity. Then, measurements were performed during three different tasks, previously described in Table 1.

Even though the printer works closed and has an exhausting system working during the printing activity to avoid emissions, the operator stands frequently near the control panel. For that reason, measurements were carried out during this task, to better know the risk of exposure to potential emissions while parts are being printed.

### 2.3. Qualitative Approach

Control Banding has been often used for studying the risk of exposure to ENM and has been suggested as a potential approach to assess the risk of exposure to incidental nanomaterials [8]. In 2021, Sousa, Silva and Arezes [13] published a review on control band, focusing the occupational exposure to incidental nanoparticles. This study provided an overview on different Control Banding approaches designed for ENM and their potential to be used for incidental nanomaterials, highlighting CB Nanotool and Stoffenmanager Nano as potential methods in this field, considering some adaptations. Therefore, Control Banding Nanotool (version 2.0) and Stoffenmanager Nano (version 1.0) were used in this case study. While both methods are control banding based, their approach is significantly different, especially regarding the inputs for the determination of bands and the risk control considerations.

CB Nanotool 2.0 was proposed in 2009 [14] and revalidated by its authors 10 years later [15]. Applying this method, it is possible to determine the risk level of a particular operation using a four-by-four matrix, based on severity and probability scores. The severity score depends on factors associated with the nanomaterial (70% of the severity score) and with the parent material (30% of the severity score). Nanomaterial (NM) factors include: Surface chemistry; particle shape; particle diameter; solubility; carcinogenicity; reproductive toxicity; mutagenicity; dermal toxicity; and asthmagen. The parent material (PM) factors are scored considering: Occupational Exposure Limit (OEL); carcinogenicity; Reproductive Toxicity; Mutagenicity; Dermal Toxicity; and Asthmagen. The second step is to reach the probability score, for which the following factors are considered: estimated amount of material used; dustiness/mistiness; number of employees with similar exposure; frequency of operation; and duration of operation. Finally, after reaching a severity and probability score, this tool leads to one of four risk levels (RL), which correspond to a certain control measure: RL 1—general ventilation; RL 2—fume hoods or local exhaust ventilation; RL 3—containment; and RL 4—seeking specialist advice.

However, Stoffenmanager Nano 1.0 is a risk-banding tool created to prioritize the risk of exposure to manufactured nano-objects and to help defining control measures [16]. This tool defines five hazard bands (A being the least hazardous until E which is the most hazardous), considering hazardous characteristics of the nano-object under study, such as particle size, water solubility, persistent fibers or other structure and classification based on data available on the nano-object or on the hazardous potential of its parental material. Four exposure bands are also determined (1 to 4, with 1 being the lowest exposure), considering nine modifying factors related to source emission, transmission, and immission (receptor): Substance emission potential, handling (activity emission potential), localized controls, segregation, dilution/dispersion, personal behavior, separation (personal enclosure), surface contamination, and respiratory protective equipment. The online tool guides the user through six steps:

- Step 1—General: Allows the user to select the source domain of potential release of nanomaterials, among four options: release of primary particles during actual synthesis; handling of bulk aggregated/agglomerated nanopowders; spraying or dispersion of a ready-to-use nanoproducts; or fracturing and abrasion of manufactured nano-objects-embedded end products.
- Step 2—Product characteristics: Includes information provided by product information sheets and/or material safety data sheets (if available), such as dustiness, moisture content, concentration, presence of fibers, and inhalation hazard.
- Step 3—Handling/process: Considers information to characterize tasks such as the way the product is handled, duration and frequency of the task, distance to the breathing zone of employees and number of employees performing the task.
- Step 4—Working area: Takes into account information on frequency of room cleaning, inspections and maintenance, as well as volume and ventilation conditions of the working room.
- Step 5—Local control measures and personal protective equipment (PPE): Includes information regarding control measures, location of the employees and type of PPE used during the task.
- Step 6—Risk assessment: Inputs of the 5 previous steps are considered to calculate the exposure-hazard-class and show the risk priority band using the risk matrix. Overall, 1 represents the highest priority and 3 the lowest priority.

### 2.4. Semi-Quantitative Approach—Proposal for a New Risk Management Method

After applying the previously mentioned qualitative and quantitative approaches in this case study, a different approach was designed. As highlighted by Sousa et al. [13], the existing qualitative and quantitative approaches have significant limitations when aiming to manage the risk of exposure to incidental nanomaterials, mainly during metal 3D printing. Therefore, in this study, a new semi-quantitative risk management tool was designed and verified.

The IN Nanotool is based on control banding and aims to enable the risk management of exposure to incidental metal nanomaterials released in AM processes. The existing control banding methods for studying the risk associated with exposure to nanomaterials in workplaces were designed for engineered nanomaterials [13]. However, there is a need to create methods to study the risk of exposure to incidental ones, since the number of workers exposed to them is significantly higher than the ones exposed to ENM [17].

Therefore, IN Nanotool was designed taking into consideration the limitations and opportunities already identified in previous studies regarding exposure to incidental nanomaterials in addition to the results of this particular case study.

## 3. Results

### 3.1. Quantitative Assessment

#### 3.1.1. On-Site Measurements

Temperature, relative humidity, and air velocity were determined to characterize the background environmental conditions of the prototyping room and the conditions near the 3D printer while it was printing, as shown in Table 2.

**Figure 1:**
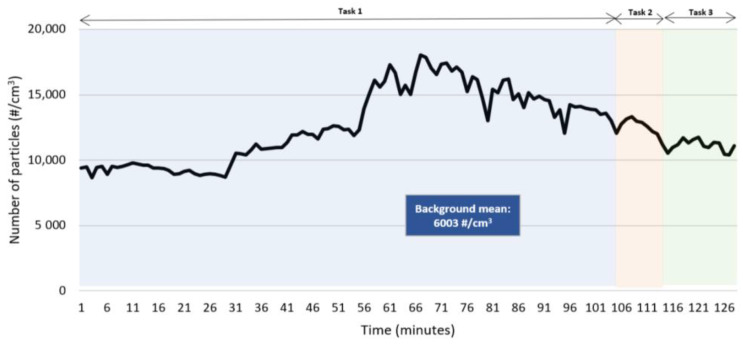
Number particle concentration (#/cm^3^) over time measured by the CPC.

The CPC provided the particle number concentration, from 10 nm to >1000 nm, during the three tasks under study, in addition to the background measurement. Table 3 indicates the mean particle number concentration for these four distinct periods. Additionally, Figure 1 illustrates how the concentration of this airborne particle changed over time during the trial.

**Figure 2:**
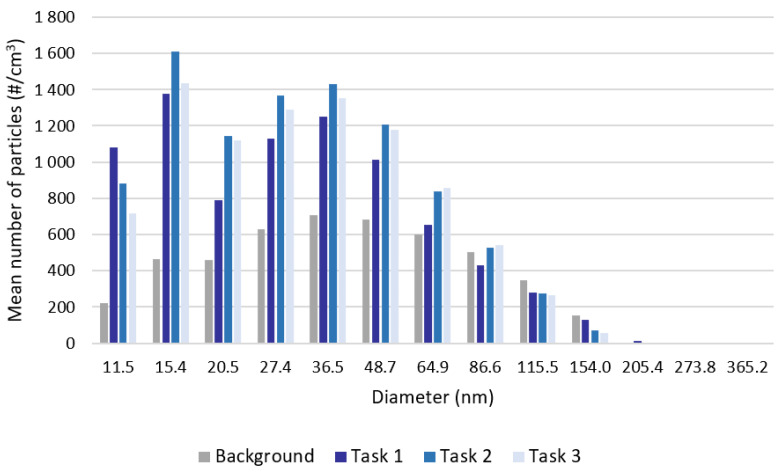
Results provided by the SMPS: Mean particle number concentration (particles/cm^3^) by particle size range.

The SMPS allowed us to better understand the potential exposure to smaller particles, by providing the size distributions from 10 to 420 nm. The corresponding results are presented in Table 4 and Figure 2.

#### 3.1.2. SEM and EDS

**Figure 3:**
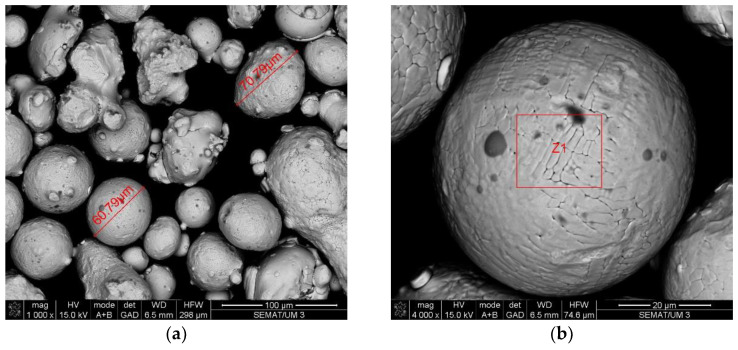
SEM analysis results: Stainless steel 316L raw powder, before any AM process. (**a**) Size and shape of particles in the sample; (**b**) image of the particle analyzed by EDS (results in Figure 4).

**Figure 4:**
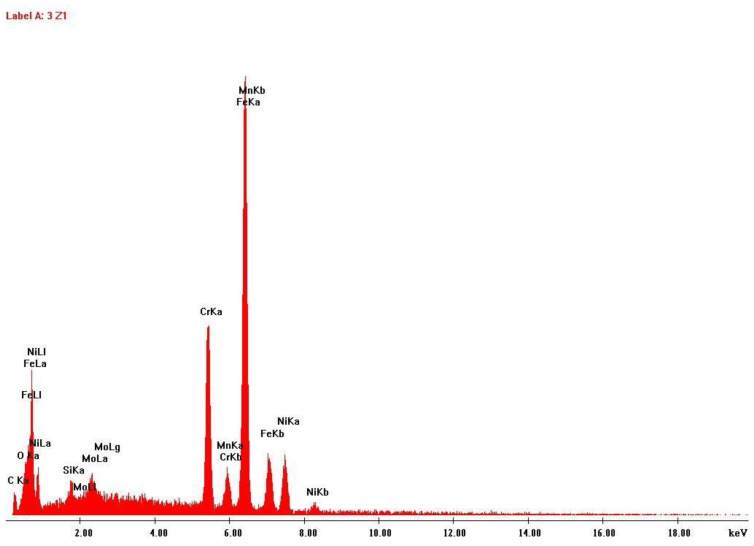
EDS analysis results: Stainless steel 316L raw powder, before any AM process.

**Figure 5:**
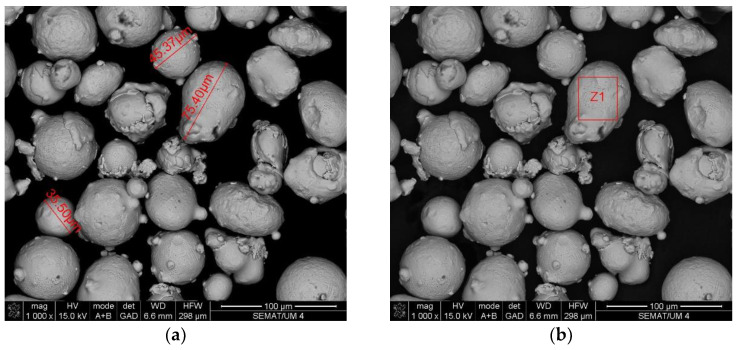
SEM analysis results: Stainless steel 316L powder after AM process. (**a**) Size and shape of particles in the sample; (**b**) image of the particle analyzed by EDS (results in Figure 6).

**Figure 6:**
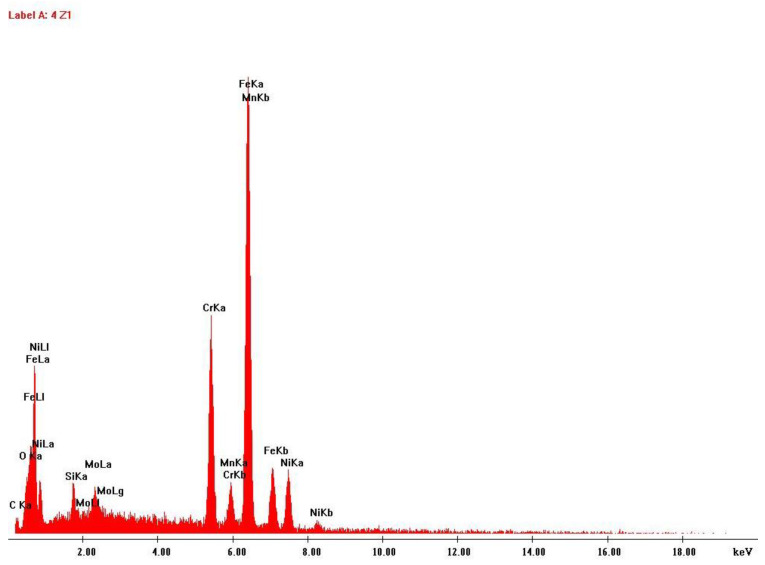
EDS analysis results: Stainless steel 316L powder after printing.

The data collection included two samples of stainless steel 316L: One of the raw powder before printing and other of the powder after the laser action, which is collected after printing to be reused in future prints. Scanning electron microscopy and energy-dispersive X-ray spectroscopy analysis were performed to these two samples, to study possible changes in size, shape and/or chemical composition. The results are shown in Figure 3, Figure 4, Figure 5 and Figure 6.

**Figure 7:**
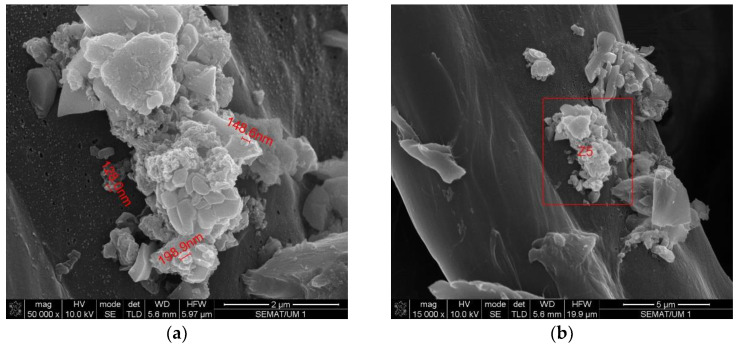
SEM analysis results: airborne sample collected on quartz filter. (**a**) Size and shape of particles in the sample; (**b**) image of the particle analyzed by EDS (results in Figure 8).

**Figure 8:**
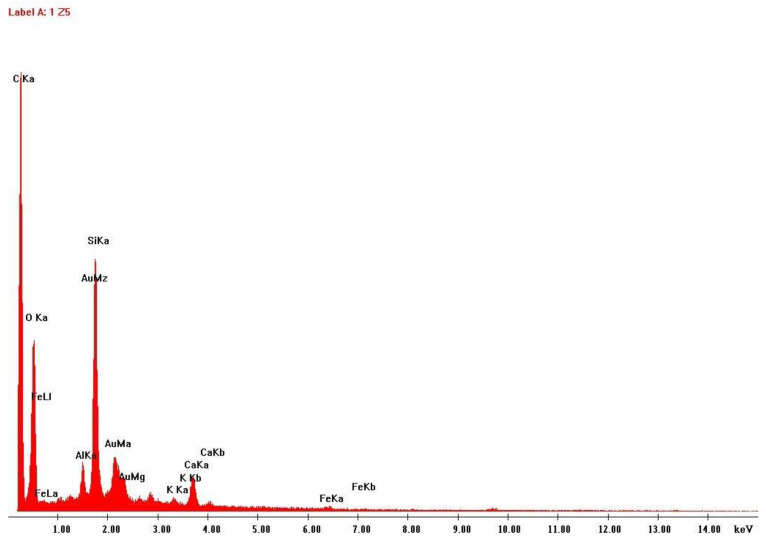
EDS analysis results: Airborne sample collected on quartz filter.

**Figure 9:**
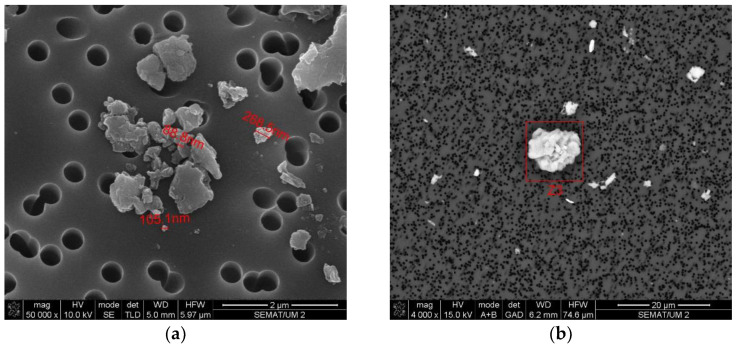
SEM analysis results: airborne sample collected on polycarbonate filter. (**a**) Size and shape of particles in the sample; (**b**) image of the particle analyzed by EDS (results in Figure 10).

**Figure 10:**
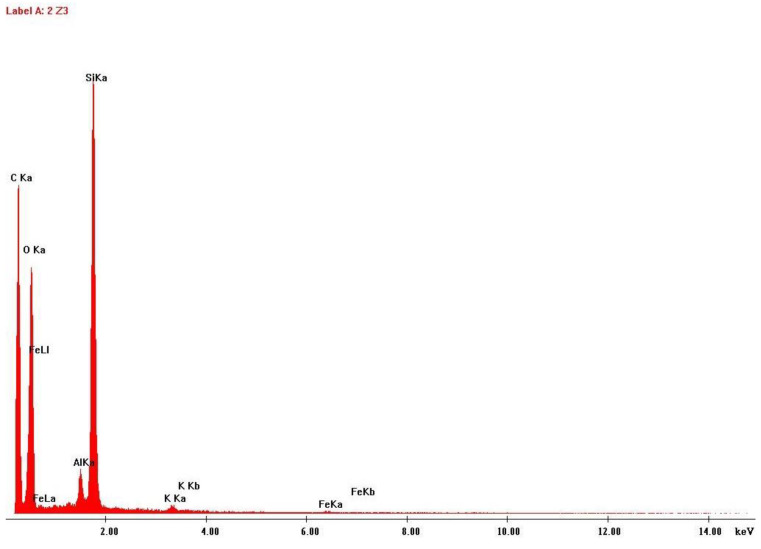
EDS analysis results: Airborne sample collected on polycarbonate filter.

To better characterize the size and shape of the particles released to the work atmosphere during this AM process, the air samples collected on the polycarbonate membrane filter and on the heat-treated quartz filter were subjected to SEM. EDS analysis was also carried out to verify the elementary composition of these samples. Figure 7, Figure 8, Figure 9 and Figure 10 illustrate these results.

### 3.2. Qualitative Assessment

#### 3.2.1. Control Banding Nanotool 2.0

As mentioned before, CB Nanotool 2.0 was one of the methods used to qualitatively assess the risk of exposure to incidental nanoparticles during the tasks understudy. Table 5 summarizes the considerations and results of the application of this qualitative method.

#### 3.2.2. Stoffenmanager Nano 1.0

The results of the application of Stoffenmanager Nano 1.0 to assess qualitatively the risk of exposure to incidental nanoparticles during the tasks understudy are in Table 6.

### 3.3. IN Nanotool—Design

#### 3.3.1. Framework

As previously mentioned, one of the main goals of this study was to design a more accurate control banding based method to manage the risk of exposure to incidental nano-scale matter in metal AM workplaces. This was only possible after studying and understanding the limitations and potential of the currently used methods.

The IN Nanotool redefined inputs by adapting them to incidental nanomaterials originating from metal powders. Additionally, this tool added quantitative data as a potential input, given the possibility to include information on shape and size of nanomaterials, taking into consideration that many authors consider this information fundamental to classify hazards [16].

The IN Nanotool defines four hazard bands, considering metal powder properties and airborne nanomaterials properties, and four exposure bands, considering materials and operation conditions and existing control measures. Then, it allows for the determination of the risk level associated with the exposure to nanomaterials during metal AM, according to previously determined hazard and exposure bands, using a four-by-four matrix. Finally, this method recommends additional control measures depending on the risk level, as an increment to the existing ones.

IN Nanotool was thought to be used by occupational safety and health (OSH) professionals, including non-experts. Therefore, it aims to be an intuitive and user-friendly tool, maintaining the necessary accuracy for an assertive risk management, guaranteeing the safety and health conditions of exposed workers. The assessment steps are described in detail on the following subsubsections.

#### 3.3.2. Hazard Band Determination

The hazard band is determined by the sum of all points from 11 different factors related to the metal powder characteristics (50 possible points out of 100) and the airborne nanomaterials characteristics (50 possible point out of 100), as summarized in Table 7.

Regarding the properties of the metal powder, the first six factors are related with the hazard classification of the powder: Carcinogenicity, reproductive toxicity, mutagenicity, dermal toxicity, inhalation toxicity and/or other significant health hazards. These properties can be verified, for example, on the second section of the material safety data sheet (MSDS) of the product (hazard identification), confirming if any of the related hazard statements are included. Other CB methods for ENM also include some of this information [14,16,18]. Regardless, IN Nanotool attempts to better catalog these hazards in different factors and also to simplify the process of classification by using as guideline the related hazard statements, according to European Classification, Labelling and Packaging (CLP) Regulation. Many authors considerer that standardized communication, such as MSDS, should be the source of hazard information, including Stoffenmanager authors [19].

There are three more factors for the characteristics of the metal powder: Lowest Occupational Exposure Limit (OEL) applicable, solubility, and average particle size. The first one is based on the CB Nanotool factor Parent Material OEL, considering that it is important to take into account the known and already established occupational exposure limits. These limits may originate from bibliography, legislation, standardization or other reliable source. Next factor, solubility, is a physicochemical property considered in most CB approaches to study exposure to ENM [20]. A material is not considered water-soluble unless the solubility limit exceeds 1 g/L or is listed as soluble or highly water-soluble. Points are given considering that even if the material is soluble does not mean there is no hazard; thus nano-specific properties are expected to be lost when particles are in solution [16]. Finally, the average particle size factor is taken into account, since the size of the primary particles is an important input for a precautionary approach [21]. The particle size can sometimes be found in the material safety data sheet of the product or in its technical sheet. Alternatively, it is possible to obtain this information by performing a SEM or TEM analysis. The points are given depending on a range of sizes, that goes from smaller than 50 µm to higher that 100 µm. Even though, in SLM technology it is very common to use metal powders with a typical particle size of 40 µm [7], there are other technologies that use different size ranges. For instance, several AM technologies use metal powder between 15 to 100 µm [22].

To complete the hazard band determination, there are two significant factors related to the properties of airborne nanomaterials: Shape and size. Shape is also an input in CB Nanotool 2.0 for the severity band of ENM [14] and it was also considered in IN Nanotool given its relevance. It can be scored considering, for example, results of a SEM or TEM analysis. Regarding size, despite the definition of nanomaterial, cells and organisms are also affected by particles whose external dimensions are bigger than 100 nm, since cells are capable of absorbing particles of up to approximately 500 nm [21]. Therefore, it is possible to assign different scores in this last factor, depending on the main size range: Lower than 100 nm, between 100 and 500 nm or higher than 500 nm. This factor can be scored considering, for example, results of a SEM or TEM analysis. If it is not possible to obtain accurate information on the shape and size of airborne matter, the IN Nanotool allows the user to assign 18.75 points to each factor, assuming it is unknown. In fact, for all 11 factors it is possible to classify the factor as unknown, giving the uncertainty in these studies.

After assigning scores to all 11 factors, the hazard band is determined depending on the sum of these points. There are four different hazard bands: low (0–25), medium (26–50), high (51–75) or very high (76–100).

#### 3.3.3. Exposure Band Determination

The exposure band is determined by the sum of all points from five distinct factors related to material operation conditions (60 possible points out of 100) and four factors associated with existing control measures (40 possible points out of 100), as presented in Table 8.

The first five factors are related with the material and operation conditions: Dustiness, frequency of operation, duration of operation per day, task characterization and estimated amount of powder used in that task. When handling a powdered material, the main factor for intrinsic emission potential is dustiness [23], therefore this is factor number one in the exposure band factors of the IN Nanotool. Points are given based on a judgment of whether the material’s dustiness is high, medium, or low. Most of these five factors are also considered in the other nano CB approaches, since they are essential to study exposure to nanomaterials [24]. In IN Nanotool, the number of employees exposure was not considered, since 3D printers usually are operated by only one or two workers, which means this is not a very relevant input to determine exposure in these workplaces.

The last four factors are related to existing control actions. Considering the already implemented control measures, it is possible to assess the actual exposure of the worker. Therefore, IN Nanotool follows a similar approach to Stoffenmanger Nano [16], which does not compromise the subsequent proposal for additional control measures that can be implemented and effectively reduce the risk.

After summing the scores of the nine factors, the exposure band is defined according to the following criteria: low if the score is under 25, medium if the score is between 26 and 50, high if between 51 and 75 or very high if the sum is 76 or higher.

#### 3.3.4. Risk Level Determination

**Figure 11:**
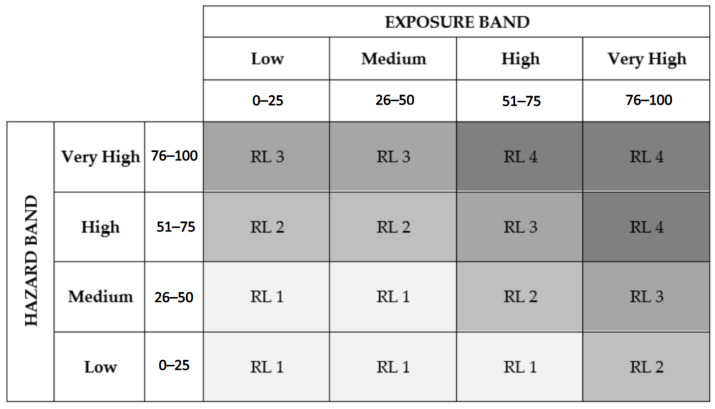
IN Nanotool risk matrix. The darker the color the higher the risk level.

After defining the hazard and exposure bands, IN Nanotool allows the user to determine the risk level using a four-by-four matrix, as commonly used in other CB strategies [25]. This risk matrix is presented in Figure 11, and it is based on the matrix of the CB Nanotool 2.0.

#### 3.3.5. Risk Control

The IN Nanotool aims not only to assess the risk of exposure to incidental metal nanomaterials, but also to help users to properly manage this risk by providing recommendations for risk control. These recommendations depend on the risk level and on the control measures already implemented. They aim to be an increment to the already existing measures. For each risk level, there is more than one recommendation. The user must analyze the options and select one (or more) that is not yet implemented and that can ideally have an impact on the higher scored factors. A new risk assessment may be performed after the implementation of the recommended controls, to validate the risk level decrease. However, when selecting the control, the user can take advantage of the tool to assess the impact of that measure in the risk level, helping to choose the more effective control measure. Table 9 shows the list of recommended additional control measures based on risk level.

### 3.4. IN Nanotool—Case Study Application

To experiment and verify the potential of IN Nanotool concept, this tool was applied to the SLM printer case study. The inputs had in consideration the MSDS and the technical sheet of the powder, the Portuguese Standard NP 1796:2014 (regarding the lowest OEL) [26], SEM results presented in Section 3.1, printer manufacturer information and in situ observation and consultation of workers. Table 10 shows the results of the application of the IN Nanotool to this case study.

## 4. Discussion

### 4.1. Quantitative Assessment

On-site measurements showed the lowest mean number particle concentration on the background trial, as expected, since the printer was not yet operating. After the AM operation started, the highest mean number particle concentration was obtained while the worker removed the part from the printer and cleaned it with a brush (task 2), as shown in Table 3. This number is very close to the one measured during the first task (printing). In reality, when analyzing Figure 1, it is possible to verify that the highest values of the number of particles occurred during the printing process, and not during the subsequent tasks. This result may be an indicator that, although the metal parts are printed in a closed chamber, there is still emission of matter during the process that may be released into the work atmosphere. In fact, the real-time measurement of air velocity near the door of the printer indicated 0.17 m/s, as shown in Table 2, endorses this possibility, since it is significantly higher than the background measurement (<0.01 m/s). Regardless this finding, several studies showing results of workplace airborne matter measurements during metal 3D printing do not consider the printing process [5,6,8]. In view of these results, further investigation is needed in this field, to verify if currently containment conditions are enough to prevent workers’ exposure to nanomaterials during printing processes, or if containment improvement is required and/or if safety-by-design measures are needed at the printer manufacturing stage.

The results of the SMPS shown in Table 4 are consistent with the ones from the CPC (Table 3). When comparing these results to the previously mentioned recommended value of 20,000 nanoparticles/cm^3^ for an 8-h exposure time (mean number of particles between 10 and 100 nm lower than 9300 particles/cm^3^ for all tasks), it is possible to conclude that the results are consistently lower, which does not mean an absence of risk. In Figure 2, it is possible to confirm that SMPS measurements indicate that the smaller particles are released during the printing activity.

Another finding of this quantitative approach, by using the EDS technology, was that there was no significant change in the chemical composition of the powder after laser action (Figure 4 and Figure 6). The same results were achieved in similar studies [7]. The results of SEM analysis to the airborne samples (Figure 7 and Figure 9) indicate the presence of agglomerates/aggregates of nanometer-scale particles, with an anisotropic shape.

This quantitative approach gives good insights on number particle concentration, size and shape of airborne matter, chemical composition, and environmental conditions.

### 4.2. Qualitative Assessment

Qualitative assessments present risk levels as a result and allow the user to access information on recommended controls. Additionally, opposite to quantitative analysis, this approach does not require access to measuring equipment.

Table 5 summarizes the application of CB Nanotool 2.0 to this case study. Since stainless steel 316L is an alloy of iron and chromium and contains a significant quantity of nickel (≈12.5%), nickel inorganic compounds’ OEL was considered as PM OEL, since it is the lowest one amount the significant components of this alloy. According to the material safety data sheet, the metal powder used is carcinogenic (H351) and skin sensitizing (H317), so PM carcinogenicity and PM dermal toxicity factors were scored as yes (this last one considering a precautionary approach). All nanomaterial related factors were classified as “unknown” since there is no information available for these airborne incidental nanomaterials. These considerations lead to a severity score of 63 (high band) for all tasks performed.

Regarding probability band, the amount of powder used in each task is similar (always more than 100 mg). So is the number of employees exposure and the frequency of the operation, thus scores were the same. Only the duration of the operation is different, so the probability score for task 1 (the longest one) is 85 (Probable band) and for task 2 and 3 the score is 70 (Likely band). According to these results, for task 1 it is recommended to seek specialist advice since risk level is the highest possible. For task 2 and 3 the recommendation is containment since the Risk Level is 3.

These results may be considered unexpected, since the highest risk level is usually associated with handling tasks, like sieving and cleaning [27]. Another observation of the CB Nanotool results is related to the recommended controls. Containment may not be adequate for task 2 and 3 since it may not be viable when carrying the part to remove it from the chamber of the machine and when removing the remains of powder.

However, Stoffenmanager Nano 1.0 lead to different results, as presented in Table 6 since it is a source-receptor model [28]. The criteria for the hazard band were the same for all tasks: dry powder with very high dustiness, small concentration of nanocomponents and unknown characteristics of the nanomaterials (concentration and inhalation hazard). In the factor related to OECD components, the option “other MNOs” was selected in the absence of another specific for incidental NM and, in the last factor, it was necessary to establish a relation between the current hazard identification and the one considered in this method, defined in Annex III of European Union Directive 67/548/EEC, which is no longer in force (replaced by CLP Regulation No 1272/2008). Hazard band E, the hazardous one, was therefore the result for the 3 tasks. In this method, hazard band E is assigned when the parental material is classified for carcinogenicity, mutagenicity, reproduction toxicity, or sensitization [16].

Concerning the exposure band, the duration of each task was considered, as well as distance to the breathing zone of the worker and specific existing local control measures for each operation. Thus, exposure band 1 was the result for task 1 (lowest band) and exposure band 2 for the other tasks. Despite the different exposure bands, the overall risk level for all tasks was RL 1 (highest priority).

Subsequent controls recommended for each task are listed in Table 6 and they are different for tasks 1 as it shows lower exposure. The recommendations for printing operation include automation of tasks and enclosure of the source, which are already implemented. It also mentions controls that are not viable for this operation, such as wetting the powder or eliminating this task, since it would compromise all the manufacturing process. For task 2 and 3, recommendations also mention already implemented controls, such as respiratory protection, and not suitable solutions, like using a spraying booth or wetting the powder.

When applying Stoffenmanager Nano 1.0 to this case study, the hazard band E was obtained for all tasks, therefore risk level 1 was the corresponding final result by default. In view of these results, it is possible to conclude that, although this method considers relevant inputs for incidental NM and considers some control measures already implemented, it is not a suitable method for metal AM workstations, since it does not differentiate the level of risk of different tasks performed and it does not provide tailored control actions aiming at the reduction of the exposure risk in these workplaces.

### 4.3. IN Nanotool

Considering all results and limitations from the previous described qualitative and quantitative approaches, IN Nanotool was designed for managing the exposure to incidental NM in metal 3D printing workstations and it was applied in this case study. The results of this application are presented in Table 10, in which it is possible to verify that the results obtained by using the IN Nanotool are significantly different from the ones achieved by the other approaches.

When analyzing the hazard band, the first six factors were provided by the properties of the metal powder present in the MSDS of the product, being clear that it is a powder with carcinogenicity and other associated health hazards. The lowest OEL criteria was the same as the one used for CB Nanotool 2.0 application. According to its MSDS the powder is water insoluble, and the average particle size range is between 20 and 53 µm. The two remaining factors to define the hazard band (shape and size) were possible to score due to the results of SEM analysis (Figure 7 and Figure 9). If these SEM results would not be available, the score of these two factors would be 18.75 (unknown), which would increase the hazard band, since a precautionary approach is intended. The hazard band obtained for all three tasks is Medium (47 points), since the material used is the same throughout all 3D printing process.

Regarding the exposure band in this case study, material and operation conditions were determined by observing the conditions in situ and consulting the organization and the workers involved. The outcome was an exposure score of 46.5 (medium band) for task 1, mainly because it was considered that there is containment of the source and high dustiness, even though the time of exposure is higher, and no PPE were used during this period. For tasks 2 and 3 the exposure score was 70, meaning the exposure band is high. In this case, although the worker uses filter mask FFP3 and protective clothing, no eye protection is used and there is no containment of the source or isolation of the worker, when dustiness is high.

Using the risk matrix from Figure 11, it is possible to conclude that the printing process represents a Risk Level 1 and the other two tasks a Risk Level 3. These results are different from the ones obtained by applying CB Nanotool 2.0 and Stoffenmanager Nano 1.0. Using IN Nanotool, distinct risk levels are obtained for considerably different operations and the results seem to support the belief that not contained manual handling processes are the ones with higher risk [26].

It should be highlighted that in this case study using IN Nanotool the highest risk level (RL4) was not assigned to any of the tasks under study. This is aligned with the quantitative results, that show that the measured number particle concentration was not high when comparing to other metal 3D printing case studies [6,8] and to previously mentioned nano reference values.

Finally, according to the IN Nanotool, additional risk control measures should be considered. Critically analyzing the recommended controls for task 1 (see Table 10), in addition to the already containment of source, mechanical ventilation can be installed in the room, the operation conditions can change (for example, by reducing the frequency and/or duration), additional PPE can be used by precaution and/or internal procedures can be improved. For tasks 2 and 3, it is possible to clean the part with a brush and to sieve the powder in a glove box or bag, to install local exhaust ventilation or fume hood and/or to change operation conditions.

## 5. Conclusions

The difficulties to manage the risk of exposure to incidental nanomaterials and the lack of information on this matter have been recently discussed and are a cause of concern. Quantitative assessments require access to specific measurement equipment and don’t provide control recommendations, requiring expert knowledge to assess and control the risk. On the other hand, limiting the risk management approach to the existing qualitative tools focused on ENM may be biased. Using those methods for incidental NM represents a significant difficulty in background data gathering, as shown in this case study.

The main objective of this study was to explore and highlight these difficulties and to design and test a tool to manage the risk of exposure to metal incidental NM in 3D printing processes. IN Nanotool redefined the inputs of CB approaches for incidental NM and added quantitative ones. Unlike quantitative approaches, this method does not necessary require special measurement equipment and it is not dependent from reference or limit values. Moreover, this method culminates in risk control recommendations, allowing to manage the risk of exposure to airborne incidental NM originated in metal AM processes, without the need to resort to a specialist. This tool was designed to enable this risk management, by providing a comprehensive and accessible approach to OSH professionals, including non-experts. However, there are limitations to this method. For instance, if the user does not have access to majority of background information, the method allows to score factors as unknown, resulting in a high risk level. This precautionary result may lead to the suggestion of exaggerated control measures in relation to the real risk. Additionally, this tool requires additional testing and further validation. Regardless of its limitations, the IN Nanotool application to the present case study led to reliable results that are more in line with the state-of-the-art, showing its potential to fill the lack of methods for incidental NM.

## Figures and Tables

**Table 1:** Description of the tasks under study.

Task 1	Supervision of printing process
Task 2	Removing the part from the printer and cleaning it with a brush
Task 3	Removing the remains of powder, sieving it for reuse and cleaning the powder container

**Table 2:** Environmental characterization provided by the thermo-hygrometer: Air velocity, room temperature and relative humidity.

Measured Parameters	Background (before Printing)	Near the Printer (Close to the Door)
Temperature [°C]	27.4	28.1
Relative Humidity [%]	54.2	53.7
Air Velocity [m/s]	<0.01	0.17

**Table 3:** Results provided by the CPC.

	Background	Task 1	Task 2	Task 3
Time [min]	15	105	8	15
Mean particle number concentration [particles/cm^3^]	6003.07	12,636.92	12,734.70	11,121.98

**Table 4:** Results provided by the SMPS: Mean particle number concentration (particles/cm^3^) by particle size.

	11.5	15.4	20.5	27.4	36.5	48.7	64.9	86.6	115.5	154.0	205.4	273.8	365.2	Total	GSD ^(1)^
Background	219.69	461.75	458.70	630.35	709.28	684.59	602.07	502.39	347.25	153.61	3.89	0.00	0.00	4773.58	40.63
Task 1	1080.31	1379.35	788.44	1129.11	1250.13	1010.95	654.65	430.89	278.75	129.33	13.35	0.00	0.12	8145.38	30.66
Task 2	883.65	1611.25	1146.33	1365.82	1430.65	1206.07	836.94	527.58	274.65	72.86	0.00	0.00	0.00	9355.81	30.27
Task 3	714.64	1436.72	1118.46	1292.11	1353.58	1177.73	856.14	543.37	266.28	54.00	0.00	0.00	0.00	8813.04	31.19

^(1)^ Geometric Standard Deviation.

**Table 5:** Results of the application of CB Nanotool version 2.0.

CB Factors	Task 1	Task 2	Task 3
PM OEL	200 µg/m^3 1^	200 µg/m^3 1^	200 µg/m^3 1^
PM Carcinogenicity	yes ^2^	yes ^2^	yes ^2^
PM Reproductive Toxicity	no	no	no
PM Mutagenicity	no	no	no
PM Dermal Toxicity	yes ^3^	yes ^3^	yes ^3^
PM Asthmagen	no	no	no
NM Surface Chemistry	unknown	unknown	unknown
NM Particle Shape	unknown	unknown	unknown
NM Particle Diameter	unknown	unknown	unknown
NM Solubility	unknown	unknown	unknown
NM Carcinogenicity	unknown	unknown	unknown
NM Reproductive Toxicity	unknown	unknown	unknown
NM Mutagenicity	unknown	unknown	unknown
NM Dermal Toxicity	unknown	unknown	unknown
NM Asthmagen	unknown	unknown	unknown
**Severity Score | Band**	**63 | High**	**63 | High**	**63 | High**
Estimated amount of material used	>100 mg	>100 mg	>100 mg
Dustiness/mistiness	high	high	high
Number of employees with similar exposure	1–5	1–5	1–5
Frequency of operation	daily	daily	daily
Duration of operation	>4 h	<30 min	<30 min
**Probability Score | Band**	**85 | Probable**	**70 | Likely**	**70 | Likely**
**Risk Level and recommended controls**	**RL 4—Seek** **specialist advice**	**RL 3—Containment**	**RL 3—Containment**

^1^ Considering the lowest OEL recommended in Portugal: Nickel inorganic compounds (Portuguese Institute of Quality, 2014). ^2^ Carc. 2, H351 according to the material safety data sheet. ^3^ Skin Sens. 1, H317 according to the material safety data sheet.

**Table 6:** Results of the application of Stoffenmanager Nano 1.0.

CB Factors	Task 1	Task 2		Task 3
Product appearance	powder	powder		powder
Dustiness	very high	very high		very high
Moisture content	dry product	dry product		dry product
Exact concentration of the nano component	unknown	unknown		unknown
Concentration	small (1–10%)	small (1–10%)		small (1–10%)
Fibers or fiber like particles in the product	no	no		no
Inhalation hazard	unknown	unknown		unknown
OECD components	other MNOs	other MNOs		other MNOs
PM with one or more of the R phrases: R40, R42, R43, R45, R46, R49, R68 ^1^	yes	yes		yes
**Hazard Band**	**E**	**E**		**E**
Task characterization	Handling of products in closed containers	Handling of products with low speed or little force		Handling of products with low speed or little force
Duration task	30–120 min/day	1–30 min/day		1–30 min/day
Frequency task	≈4 to 5 days/week	≈4 to 5 days/week		≈4 to 5 days/week
Distance head-product (breathing zone)	>1 m	<1 m		<1 m
More than one employee performing the task simultaneously	no	no		no
Room cleaned daily	yes	yes		yes
Inspections and maintenance of machines/ancillary equipment performed at least monthly	no	no		no
Volume of the working room	100–1000 m^3^	100–1000 m^3^		100–1000 m^3^
Ventilation of the working room	Mechanical and/or natural ventilation	Mechanical and/or natural ventilation		Mechanical and/or natural ventilation
Local control measures	Containment of the source with local exhaust ventilation	none		none
The employee is situated in a cabin	no	no		no
Personal Protective Equipment used	none	Filter mask P3 (FFP3)		Filter mask P3 (FFP3)
**Exposure Band**	**1**	**2**		**2**
**Risk Level**	**RL 1—Highest priority**	**RL 1—Highest priority**		**RL 1—Highest priority**
**Recommended controls**	▪ Product elimination ▪ Task elimination ▪ Product substitution ▪ Automation of tasks ▪ Enclosure of the source ▪ Local exhaust ventilation ▪ Enclosure of the source in combination with local exhaust ventilation ▪ Wetting of powders/substance ▪ Applying glove boxes/bags ▪ Use of a spraying booth ▪ Use of work cabins with clean air supply ▪ Use of work cabins without clean air supply ▪ Respiratory protection		▪ Product elimination ▪ Task elimination ▪ Process adaptations ▪ Product substitution ▪ Automation of tasks ▪ Enclosure of the source Local exhaust ventilation ▪ Enclosure of the source in combination with local exhaust ventilation ▪ Wetting of powders/substance ▪ Applying glove boxes/bags ▪ Use of a spraying booth ▪ Use of work cabins with clean air supply ▪ Use of work cabins without clean air supply ▪ Respiratory protection	

^1^ Defined in Annex III of European Union Directive 67/548/EEC, no longer in force; replaced by CLP Regulation No 1272/2008.

**Table 7:** Hazard factors and points per factor.

Metal Powder Characteristics									
**1. Powder Carcinogenicity:** score is assigned based on whether the material is carcinogenic or not. It is possible to confirm this information on the material safety data sheet, for example, by checking if any of these hazard statements are included in its hazard identification: H350, H351 (according to CLP Regulation).									
yes: 6			no: 0				unknown: 4.5		
**2. Powder Reproductive Toxicity:** score is assigned based on whether the material is a reproductive hazard or not. It is possible to confirm this information on the material safety data sheet, for example, by checking if any of these hazard statements are included in its hazard identification: H360, H361, H362 (according to CLP Regulation).									
yes: 6			no: 0				unknown: 4.5		
**3. Powder Mutagenicity Toxicity:** score is assigned based on whether the material is a mutagenic or not. It is possible to confirm this information on the material safety data sheet, for example, by checking if any of these hazard statements are included in its hazard identification: H340, H341 (according to CLP Regulation).									
yes: 6			no: 0				unknown: 4.5		
**4. Powder Dermal Toxicity:** score is assigned based on whether the material is a dermal hazard or not. It is possible to confirm this information on the material safety data sheet, for example, by checking if any of these hazard statements are included in its hazard identification: H310, H311, H312 (according to CLP Regulation).									
yes: 6			no: 0				unknown: 4.5		
**5. Powder Inhalation Toxicity:** score is assigned based on whether the material is toxic if inhaled or not. It is possible to confirm this information on the material safety data sheet, for example, by checking if any of these hazard statements are included in its hazard identification: H330, H331, H332, H333 (according to CLP Regulation).									
yes: 6			no: 0				unknown: 4.5		
**6. Other health hazards of the powder:** score is assigned based on other hazards of the material besides the ones already scored in factors 1 to 5. It is possible to confirm this information on the material safety data sheet, for example, by checking if any hazard statement starting with H3 is included in its hazard identification (besides the ones already mentioned in factors 1 to 5).									
yes: 4			no: 0				unknown: 3		
**7. Lowest OEL applicable to powder [µg/m^3^]:** a different score is given depending on the lowest OEL defined for the metal powder’s components.									
<100 µg/m^3^: 8	100–1000 µg/m^3^: 4		1001–10,000 µg/m^3^: 2		>10,000 µg/m^3^: 0				unknown: 6
**8. Powder Solubility:** score is given depending on the water-solubility of the material, considering it is soluble if the solubility higher than 1 g/L. If this property is unknown, 3 points are given.									
insoluble (<1 g/L): 4			soluble (>1 g/L): 0			unknown: 3			
**9. Powder Average particle size [µm]:** the score is assigned according to the available information or analyzes performed. If unknown, 3 points are given.									
<50 µm: 4		50–1000 µm: 3		>100 µm: 1				unknown: 3	
**Airborne nanomaterials characteristics**									
**10. Shape:** the score is assigned according to available information, for example, to SEM or TEM analyzes results, considering the most common shape verified. If unknown, 18.75 points are given.									
tubular, fibrous: 25		anisotropic: 12.5		compact/spherical: 6.25				unknown: 18.75	
**11. Size:** the score is assigned according to available information, for example, to SEM or TEM analyzes results, considering the main size of airborne materials. If unknown, 18.75 points are given.									
<100 nm: 25		100–500 nm: 12.5		>500: 6.25				unknown: 18.75	

**Table 8:** Exposure factors and points per factor.

Operation Conditions						
**1. Powder Dustiness:** points are provided based on a judgment of whether the material’s dustiness is high, medium, or low. If unknown, 11.5 points are given.						
high: 15		medium: 10		low: 5		unknown: 11.25
**2. Frequency of operation:** points are provided depending on the regularity of the procedure.						
daily: 10	weekly: 5		monthly: 2.5		>monthly: 0	unknown: 7.5
**3. Duration of operation (per day):** score is assigned based on the daily time dedicated to the operation.						
>4 h: 10	1–4 h: 5		30–60 min: 2.5		<30 min: 0	unknown: 7.5
**4. Task characterization:** points are provided based on a judgment of whether the quantity of dust generated and dispersed during the task is large, low or negligible during manual handling. If there is no manual handling or it is performed in a closed container (for example printing operation in a closed printer), 0 points are assigned to this factor.						
manual handling the powder where large quantities of dust are generated and dispersed: 15		manual handling the powder where low quantities of dust are generated and dispersed: 10		manual handling the powder where negligible quantities of dust are generated and dispersed: 5		no manual handling or handling in closed containers: 0
**Existing control measures**						
**5. Working room control measures:** points are provided by confirming on-site ventilation conditions.						
no general ventilation: 10		natural ventilation: 5		mechanical ventilation (alone or combined with natural ventilation): 0		unknown: 7.5
**6. Source control measures:** score is given by confirming the control measures on the source of emissions.						
no control measures at the source: 15		use of a product that limits the emission: 10		local exhaust ventilation or fume hood: 5		containment of the source or glove box or glove bag: 0
**7. Preventive procedures:** score is assigned according to the existing cleaning and maintenance routines.						
room cleaned daily and printer maintenance performed at least monthly: 0			cleaning and maintenance procedures less frequent than previous option: 10			unknown: 7.5
**8. Worker related control measures:** points are chosen considering the personal protective equipment (PPE) used by the worker.						
The worker does not work in a separate room/cabin and does not use any PPE: 5						
The worker uses eye protection and/or protective clothing (including gloves): 4						
The worker uses filter mask P2/FFP2: 4						
The worker uses filter mask P2/FFP2 and protective clothing (including gloves) or eye protection: 3						
The worker uses filter mask P2/FFP2, protective clothing (including gloves) and eye protection: 2.5						
The worker uses filter mask P3/FFP3: 3						
The worker uses filter mask P3/FFP3 and protective clothing (including gloves) or eye protection: 2.5						
The worker uses filter mask P3/FFP3, protective clothing (including gloves) and eye protection: 1						
The worker uses powered/supplied air respirator: 1						
The worker uses powered/supplied air respirator and protective clothing (including gloves) or eye protection: 0.5						
The worker uses powered/supplied air respirator, protective clothing (including gloves) and eye protection: 0						
The worker works in a separate room/cabin with independent ventilation system: 0						

**Table 9:** Recommended additional control measures based on risk level.

Risk Level	Total Score	Recommended Additional Control Measures Based on Risk Level
RL 4	151–200	Seek specialist advice Product replacement Task elimination or automatization Containment/Glove box/Glove bag Worker isolation (separate room/cabin)
RL 3	101–150	Task elimination or automatization Containment/Glove box/Glove bag Worker isolation (separate room/cabin) Local exhaust ventilation or fume hood Change operation conditions
RL 2	51–100	Worker isolation (separate room/cabin) Local exhaust ventilation or fume hood Change operation conditions Mechanical ventilation Change Personal Protective Equipment
RL 1	≤50	Change operation conditions Mechanical ventilation Change Personal Protective Equipment Improve internal preventive procedures

**Table 10:** Results of the application of the IN Nanotool.

CB Factors	Task 1	Task 2	Task 3
Powder Carcinogenicity	yes ^1^	yes ^1^	yes ^1^
Powder Reproductive Toxicity	no	no	no
Powder Mutagenicity	no	no	no
Powder Dermal Toxicity	no	no	no
Powder Inhalation Toxicity	no	no	no
Other Hazards of the powder	yes ^2^	yes ^2^	yes ^2^
Lowest OEL applicable to powder	200 µg/m^3 3^	200 µg/m^3 3^	200 µg/m^3 3^
Powder Solubility	insoluble	insoluble	insoluble
Powder Average particle size	<50 µm	<50 µm	<50 µm
Airborne NM Shape	anisotropic	anisotropic	anisotropic
Airborne NM Size	100–500 nm	100–500 nm	100–500 nm
**Hazard Score | Band**	**47 | Medium**	**47 | Medium**	**47 | Medium**
Powder Dustiness	high	high	high
Frequency of operation	daily	daily	daily
Duration of operation (per day)	1–4 h	<30 min	<30 min
Task characterization	No manual handing	Manual handling the powder where large quantities of dust are generated and dispersed	Manual handling the powder where large quantities of dust are generated and dispersed
Estimated amount powder used	100–1000 g	100–1000 g	100–1000 g
Local control measure— Working room	Natural ventilation	Natural ventilation	Natural ventilation
Local control measures— Source	Containment of the source	No control measures at the source	No control measures at the source
Local control measures— Preventive procedures	Room cleaned daily and printer maintenance performed at least	Room cleaned daily and printer maintenance performed at least	Room cleaned daily and printer maintenance performed at least
Local control measures— Worker	The worker uses protective clothing	The worker uses filter mask P3/FFP3 and protective clothing	The worker uses filter mask P3/FFP3 and protective clothing
**Exposure Score | Band**	**46.5 | Medium**	**70 | High**	**70 | High**
**Risk Level**	**RL 1**	**RL 3**	**RL 3**
**Recommended controls**	▪ Change operation conditions ▪ Mechanical ventilation ▪ Change Personal Protective Equipment ▪ Improve internal preventive procedures	▪ Task elimination or automatization ▪ Containment/Glove box/Glove bag ▪ Worker isolation (separate room/cabin) ▪ Local exhaust ventilation or fume hood ▪ Change operation conditions	

^1^ Carc. 2, H351 according to the material safety data sheet. ^2^ Skin Sens. 1, H317 and Stop RE 1, H372 according to the material safety data sheet. ^3^ Considering the lowest OEL recommended in Portugal: Nickel inorganic compounds (Portuguese Institute of Quality, 2014).

## Data Availability

No new data were created or analyzed in this study. Data sharing is not applicable to this article.
